# Horizontal functional gene transfer from bacteria to fishes

**DOI:** 10.1038/srep18676

**Published:** 2015-12-22

**Authors:** Bao-Fa Sun, Tong Li, Jin-Hua Xiao, Ling-Yi Jia, Li Liu, Peng Zhang, Robert W. Murphy, Shun-Min He, Da-Wei Huang

**Affiliations:** 1 Key Laboratory of Zoological Systematics and Evolution, Institute of Zoology, Chinese Academy of Sciences, Beijing 100101, China; 2 CAS Key Laboratory of Genomics and Precision Medicine, Beijing Institute of Genomics, Chinese Academy of Sciences, Beijing 100101, China; 3 Institute of Plant Protection, Henan Academy of Agricultural Sciences; 4 Network & Information Center, Institute of Microbiology, Chinese Academy of Sciences, Beijing 100101, China; 5 Department of Natural History, Royal Ontario Museum, Toronto, Ontario, Canada; 6 College of Plant Protection, Shandong Agricultural University, Tai’an, Shandong 271018, China

## Abstract

Invertebrates can acquire functional genes via horizontal gene transfer (HGT) from bacteria but fishes are not known to do so. We provide the first reliable evidence of one HGT event from marine bacteria to fishes. The HGT appears to have occurred after emergence of the teleosts. The transferred gene is expressed and regulated developmentally. Its successful integration and expression may change the genetic and metabolic repertoire of fishes. In addition, this gene contains conserved domains and similar tertiary structures in fishes and their putative donor bacteria. Thus, it may function similarly in both groups. Evolutionary analyses indicate that it evolved under purifying selection, further indicating its conserved function. We document the first likely case of HGT of functional gene from prokaryote to fishes. This discovery certifies that HGT can influence vertebrate evolution.

Horizontal gene transfer (HGT), also termed lateral gene transfer, is the transfer of DNA between organisms outside of traditional reproduction. It often results in adaptive gains of novel genes and traits^1^,^2^. Large numbers of HGTs have been identified in prokaryotes and such events constitute an important evolutionary force that modulates the evolution of prokaryote genomes^3^,^4^,^5^,^6^,^7^. This is particularly evident when the acquisition confers a selective advantage to the host, such as antibiotic resistance^8^.

Previously, HGT was thought to play only a minor role in the evolution of animal genomes because heritable changes must enter the germline, and separation of germline cells from the soma expectedly leads to low frequencies of HGT. The rapid expansion of newly sequenced animal genomes allows for the identification of additional examples of HGT, which may, in fact, be a far more important source of novel gene evolution than originally thought^2^,^9^. Genes of prokaryotic, fungal or botanical origin exist in bdelloid rotifers^10^,^11^, tunicates^12^, jelly fish^13^, starlet sea anemones^14^, nematodes^15^,^16^, and a variety of insects^17^,^18^,^19^. HGT can contribute to the adaptive potential of the recipient and some lower animals well illustrate this. For example, numerous HGT events have occurred during the evolution of plant parasitic nematodes and such transfers likely allowed the free-living ancestral nematodes to exploit plants as a new ecological niche^15^,^16^. HGT of carotenoid biosynthetic genes from fungi has endowed aphids with the ability to produce red body colour, based on which they can vary from between green and red to avoid predators and parasites^20^. In most cases, acquired genes seem to offer novel functions. Subsequent rounds of gene duplication may expand such functional gains^1^.

Little well-documented evidence supports cross-kingdom HGTs of encoding sequences to vertebrates. Although the original analysis of the human genome identified a considerable number of protein-coding genes of possible bacterial origin^21^, further study found them to be phylogenetic artefacts^22^,^23^. Evidence for the HGT of glyoxylate cycle enzymes, which may have been acquired by non-placental vertebrates, was also controversial, because the presumptive origin of the gene was uncertain and gene-loss can explain the occurrence^24^,^25^. Although HGT has been postulated to occur in primates, flies and nematodes^26^, it remains unknown in early vertebrates, such as fishes.

Herein, we show that fishes possess one gene transferred from marine bacteria. Gene expression evidence provides strong support of its functionality. These results provide the first credible evidence for functional gene transfer from bacteria to fishes.

## Results

### One gene in fishes derived from marine bacteria

**Figure 1:**
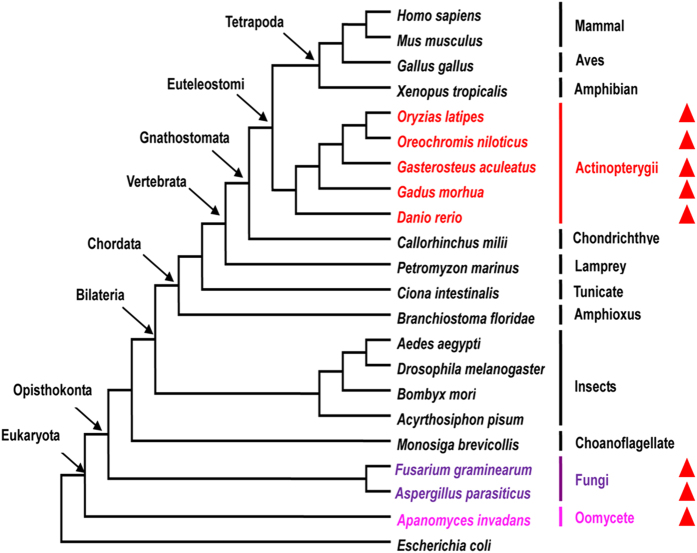
Species-tree for representative eukaryotes and the distributions of XP_001335286.1 homologs among the taxa. Representative eukaryotic clades were selected from among many to simplify depiction of the distributions of the homologs. Bacteria was used as the outgroup. Genes that were most evenly sampled across major clades were used to construct the phylogeny with ML methods. Red triangles indicate species that possess XP_001335286.1 homologs.

**Figure 2:**
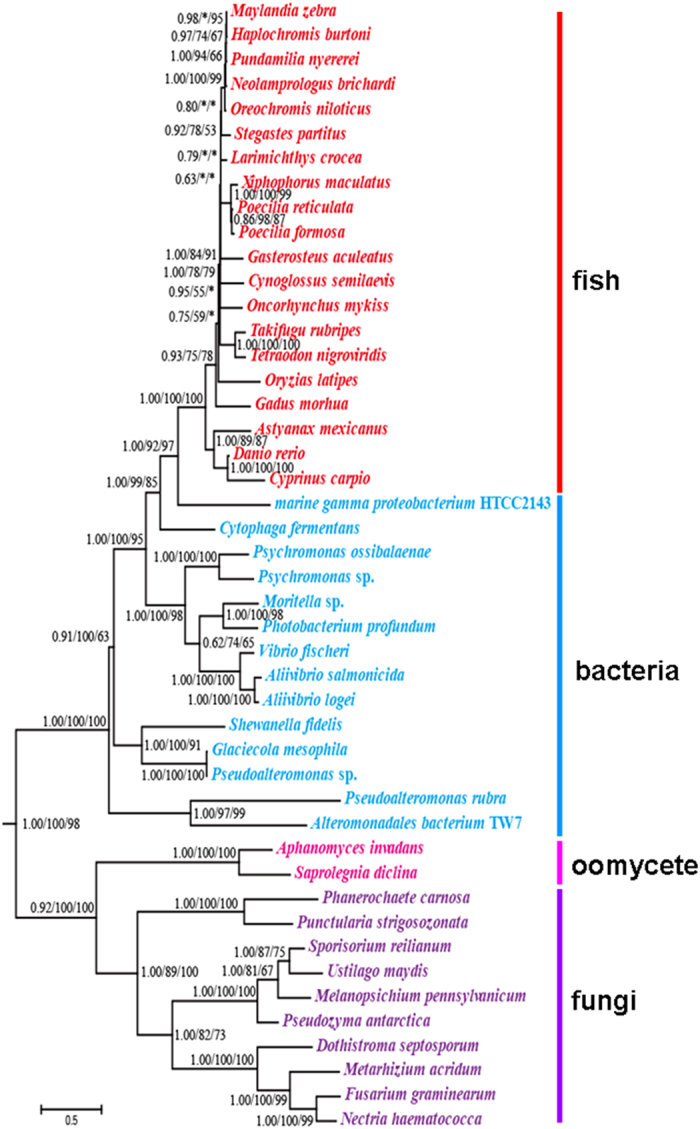
Phylogenetic trees of XP_001335286.1 homologs. The Bayesian unrooted tree (shown) is virtually identical to the ML and NJ trees. Nodal support values ≥50 given as BI/ML/NJ. Asterisks (*) indicate support values <50. Scale bar indicates substitutions per site.

**Figure 3:**
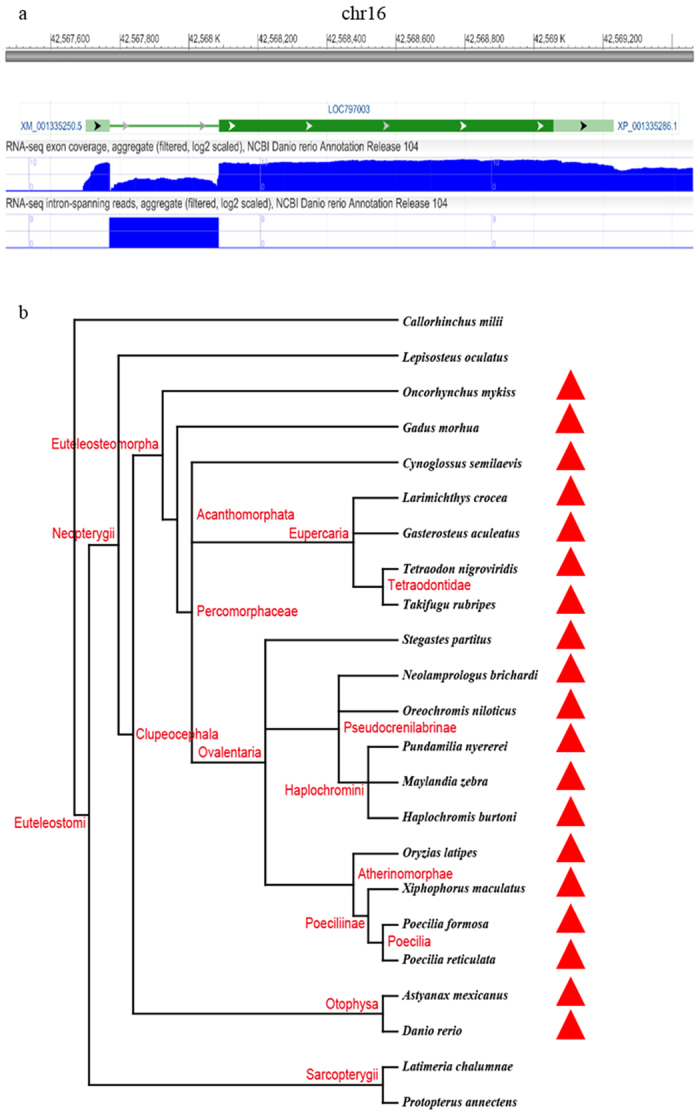
Physical maps of XP_001335286.1 in *Danio rerio* and its homologs distribution in fishes. (**a**) Physical maps of XP_001335286.1 in *D. rerio*. Arrows denote gene orientation. (**b**) Phylogeny indicates the general relationship of these fishes based on the NCBI Taxonomy information. Red triangles indicate species that possess XP_001335286.1 homologs.

Using annotated protein sequences of *Danio rerio* as input queries, a series of stringent filters served to identify promising HGTs (Supplementary Figure 1). After comparison with the proteins of other animals in RefSeq and non-redundant (NR) databases, 2766 fish-specific proteins were retained for subsequent analysis. Sixteen of these protein sequences had high similarity to non-animals, and horizontally transferred genes may have encoded these proteins. Subsequent phylogenetic analyses identified one protein with RefSeq ID XP_001335286.1 located on chromosome 16 in *D. rerio* as being potentially obtained via HGT. Next, we used either BLASTP or TBLASTN^27^ to compare this protein sequence to proteins, genomes and expressed sequences in the following databases: NR, RefSeq, Ensembl, UCSC, Expressed sequence tags (EST), Transcriptome Shotgun Assembly (TSA), and others. The results further verified its patchy phylogenetic distribution. Outside of fishes the gene had few homologues in animals, yet it was widespread in bacteria and some fungi (Fig. 1). Among non-animals, it had highest similarity with marine bacteria (Supplementary Table 1). Phylogenetic analysis clustered it with bacteria with strong support (BPP >95%; ML and NJ BS >90%) (Fig. 2). It was more likely that the transfer occurred from bacteria to fish than vice versa, assuming parsimony. A physical map of gene XP_001335286.1 in *D. rerio* documented its presence (Fig. 3a). Another gene with Refseq ID NP_001018555.1 identified initially as presumptive horizontally transferred and with top hits in non-animals, clusters with cyanobacteria well (Supplementary Figure 2). However, its phyletic positioning contrasts with HGT from cyanobacteria to eukaryotes and is more indicative of HGT in the opposite direction, for cyanobacteria embedded within a larger group of eukaryotes and did not cluster with other bacteria species. Herein we mainly study HGT in fishes, so we only focused on XP_001335286.1 in subsequent analyses.

To exclude the possibility of contamination by bacterial DNA, we confirmed the occurrence of the transferred XP_001335286.1 on multiple fish genomes. We also confirmed that native sequences were physically linked to putative horizontally transferred genes^28^. The transferred gene contained introns with splice-sites confirmed by RNA-seq data in fishes (Fig. 3a). This also precluded bacterial contamination. Finally, available RNA-seq data supported its expression in transcriptomes (Fig. 3a). Taken together, these analyses provided credible evidence that this horizontally transferred gene was integrated into the genome of fishes and did not represent a bacterial contamination.

### Antiquity of the transfer event

Transferred XP_001335286.1 had homologs in most of the sequenced fish genomes. It had greater than 70% similarities with other fishes, and these similarities were statistically highly significant with expectation values (E-values) ranging from 3e^−144^ to 0 (Supplementary Table 1). The levels of significance indicated homology. Their greater similarity among fishes than to bacteria ruled out the possibility of multiple independent HGTs (Supplementary Table 1). Finally, its homologs in fishes located in the same clade adjacent to the potential bacterial donor, further confirming it had a single, ancient transfer (Fig. 2).

Homologs of XP_001335286.1 were not detected in the genomes of basal fishes (*Callorhinchus milii* and *Lepisosteus oculatus*). All fishes with this gene belonged to Clupeocephala (Fig. 3b). Elopomorpha (tarpons, bonefishes, eels and relatives), Osteoglossomorpha (bony tongues) and Clupeocephala (all remaining teleosts), constitute the major extant teleosts of early origin^29^. Because few genomes were sequenced from the two former groups, we used TBLASTN to compare the protein against their available ESTs, TSAs and RNA-seq data, homologs were not found. This failure suggested the transfer likely occurred after emergence of Clupeocephala.

### Conserved functions and developmentally regulated expression of HGTs

**Figure 4:**
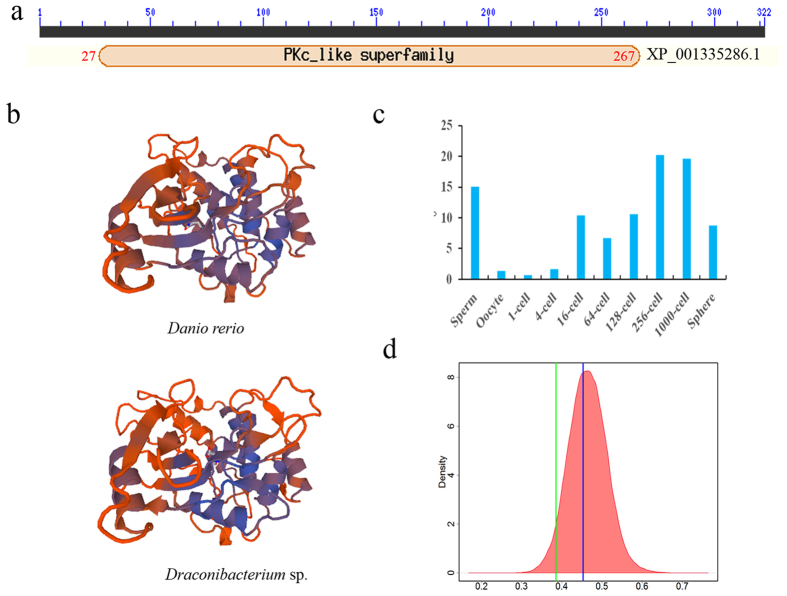
Features and expression of transferred gene in *Danio rerio*. (**a**) Domains of transferred gene coding protein XP_001335286.1. (**b**) Tertiary structures of transferred gene modelled by SWISS- MODEL server. (**c**) Expression levels of transferred gene in *D. rerio* along with developmental stages. Normalized levels of expression were estimated by count of mapped reads to this gene per kilobase of sequence length per million library reads (RPKM). (**d**) GC content distribution of transferred gene. Red area indicates the density distribution of GC content for *D. rerio* mRNA. Blue and green lines indicate the GC content of transferred gene in *D. rerio* and putative donor, respectively.

The gene transferred to fishes was an uncharacterized protein in the RefSeq database. We investigated its possible functions using bioinformatics because no prior analyses or experiments existed. Its bacterial homologs annotated as choline kinase and phosphotransferase. Pfam and SMART analyses revealed it contained the catalytic domain PKc_like super family (cl21453), also named EcKinase, and Ecdysteroid kinase (cl17738). This constituted a catalytic domain of the Protein Kinase superfamily (amino acids 27–267) (Fig. 4a). Fishes and bacteria exhibited conserved protein sequence motifs in their domain regions. This conservation suggested that the protein functioned similarly in bacteria and fishes. Although no data document the functional roles that the protein plays, it appeared to be involved in the production of enzymes needed by fishes.

We explored the cellular locations of the transferred protein using SignalP^30^, TargetP^31^, TMHMM^32^ and WoLFPSORT^33^. It had no signal peptides or transmembrane helices. WoLFPSORT predicted a cytoplasmic occurrence for it. Structure analyses by the SWISS-MODEL indicated that it had similar structures in fishes and the putative donors (Fig. 4b). Thus, it possibly played similar roles in fishes and bacterial donors.

We used *D. rerio* as a representative fish to investigate expression profiles of the transferred gene in different developmental stages. Gene XP_001335286.1 was expressed in most stages, except for oocyte and the 1-cell stage. Overall, it was highly expressed in sperm and its level of expression along increased with development up to the 1k-cell stage, indicating probable involvement in development (Fig. 4c).

Due to the absence of experimentation, we could not describe the specific function of this transferred gene. Regardless, its expression in several development stages suggested it still had important functions.

### Amelioration and purifying selection of HGTs

Amelioration, the process by which the DNA composition of the acquired gene becomes homogenized to match the composition of the recipient genome^34^, appeared to have occurred. The GC content of transferred gene was most similar to that of the fishes’ genomes, and distinct from the most probable putative donor bacterial genomes (Fig. 4d). All four indices of codon usage bias showed similar patterns of GC3s and GC content (Supplementary Figure 3). Compositional analyses showed other fishes were similar to *D. rerio* for it.

Likelihood ratio tests can identify strong selection in the evolution of genes^35^. Whereas positive selection often associates with new functions, purifying selection serves to maintain conserved functions. Global nonsynonymous (Ka)/synonymous (Ks) value of omega for XP_001335286.1 was 0.08845. Values of less than 1 have generally indicated purifying selection on gene sequences. Site-models failed to find signals of positive selection in the homologs as did branch and site models. Overall, our results suggested that purifying selection was the main driving force in the evolution of horizontally transferred XP_001335286.1. This supported the idea that this gene has conserved functions.

## Discussion

Our study is the first indication of HGT from bacteria to fishes. The phyletic distribution of the gene indicates one event following emergence of the Clupeocephala. Most new eukaryotic genes and traits originate from gene duplications where mutations subsequently modify one copy to achieve neofunctionalisation^1^. In comparison, HGT appears to be rare and to play an insignificant role in eukaryotes, especially in complex higher vertebrates^1^,^2^. The absence of a well-documented cross-kingdom HGT in vertebrates is not surprising. No mechanism explains the transfer of intact genes from non-animals to vertebrates. Such transfer may be difficult because vertebrate genes are packaged within organelles (principally the nucleus) and, therefore, they are less accessible than genes of bacteria^1^,^36^,^37^. Although HGT may occur in vertebrates^26^, gene loss, which cannot be ruled out completely, may also explain the observations.

Our analyses involving multiple databases and three tree-building methods to identify possible HGT from prokaryotes to fishes. Many fish genomes contain this horizontally transferred gene. A previous study reported an intriguing case of intra-domain HGT in fishes; a subset of antifreeze proteins (AFPs) appear to have evolved once from a lectin-like protein, and then spread by HGT between distantly related species of fishes^38^,^39^. Notwithstanding, the marine environment provides fishes an opportunity to obtain genes from environmental bacteria due to their sympatry. The urochordate *Ciona intestinalis* apparently acquired a cellulose synthase gene from Cyanobacteria^40^ and similar HGTs may also have occurred in fishes or ancestral vertebrates that have a close evolutionary relationship to *C. intestinalis.* Many aquatic organisms display HGT events^11^,^12^,^41^,^42^,^43^ and a high frequency of HGT occurs in the oceans^44^. Thus, HGT is not unexpected in fishes.

Of note, another protein within fishes, Refseq ID NP_001018555.1, its phylogenetic tree indicated one possible transfer event from vertebrates to cyanobacteria. Transfer of genes from bacteria to vertebrates is reported uncommonly, while report of functional gene transfer from vertebrates to cyanobacteria is especially rare. NP_001018555.1 may be one of the typical cases of this.

As an alternative explanation to bacteria–fish HGT, gene-loss may explain the pattern. However, this scenario requires multiple wide-scale losses in invertebrates and in vertebrates. Clearly, the origin of this gene does not date to early stages of the origin of metazoans, such as various groups of invertebrates. Further, the gene-loss hypothesis cannot explain the remarkable sequence identity between fishes and their candidate donors. It cannot explain the clustering of transferred genes with bacteria on well-supported nodes in the gene-tree. Therefore, the most plausible explanation for presence of XP_001335286 homologs in fishes is HGT from bacteria.

Though the absence of functional studies for this transfer gene hinders functional annotation, our results indicate that it contains conserved domains and similar structures in recipient fishes and their putative bacterial donors. Its expression patterns suggest involvement in development.

Analyses of the recipient horizontally transferred genes can indicate amelioration and adaptation^45^. Amelioration of a transferred gene may relate to efficient functioning in the recipient’s genome. For functional efficiency, a transferred gene needs to adjust its nucleotide compositions to match those of the new genome. The extent of amelioration also coincides with the antiquity of a transfer because fine tuning to the genomic background of the recipient requires much time. In fishes, the gene appears to have experienced purifying selection, which indicates its conserved functions. Ka/Ks values relate to protein function such that low values indicate prior purifying selection and possible functional constraints^46^.

Overall, our results provide the first probable evidence of HGT from bacteria to fishes. The transfer involves the acquisition of functions. This HGT may make significant contribution to the evolution of fishes. Further investigations will lead to a more complete understanding of the biological functions of this gene.

## Methods

### Data collection and database construction

We retrieved 43138 RefSeq proteins of the zebrafish, *Danio rerio*, and their genomic data from the National Center for Biotechnology Information (NCBI) (http://www.ncbi.nlm.nih.gov/). All protein sequences in RefSeq and non-redundant (nr) database (last accessed August 25, 2014), containing proteins of a wide diversity of eukaryote and prokaryote taxa, were also downloaded from NCBI. The corresponding taxonomy was retrieved from http://www.ncbi.nlm.nih.gov/taxonomy. In addition to *D. rerio*, sequence data were also downloaded for the following fishes: *Oreochromis niloticus, Poecilia reticulate, Neolamprologus brichardi, Pundamilia nyererei, Maylandia zebra, Poecilia formosa, Haplochromis burtoni, Stegastes partitus, Xiphophorus maculates, Oncorhynchus mykiss, Cynoglossus semilaevis, Takifugu rubripes, Tetraodon nigroviridis, Astyanax mexicanus*, *Lepisosteus oculatus*, *Oryzias latipes*, *Callorhinchus milii*, *Larimichthys crocea*, *Cyprinus carpio*, *Lethenteron camtschaticum*. Data for *Gasterosteus aculeatus*, *Gadus morhua*, *Petromyzon marinus*, *Xenopus tropicalis* and *Latimeria chalumnae* were download from Ensembl (http://www.ensembl.org/) and data for *Xenopus laevis* were download form *Xenopus* database (http://www.xenbase.org/). RNA-Seq data of the African lungfish (*Protopterus annectens*) were retrieved from the Sequence Read Archive of NCBI under accessions SRR505721–SRR505726. Transcriptome assemblies and putative ORF predictions were performed with Trinity^47^. We used TBLASTN to compare candidate HGTs to Reference of Genome sequences, Expressed Sequence Tag (EST) and Transcriptome Shotgun Assembly (TSA) databases in NCBI to obtain the possible homologous genes^27^.

We constructed two local proteome databases for the integrated proteins. One database contained 1233189 proteins of animals and the other one was comprised of 10605769 proteins excluding animals.

### Screening for candidate HGTs

We used a comprehensive bioinformatic pipeline involving multiple steps to identify HGT candidates in fishes (Supplementary Figure 1). The genome of *D. rerio* was used to represent fishes due to its high level of integrity and accuracy, which was much better than those of other fishes. Therefore, we mainly focused on proteins of *D. rerio* to detect candidate HGT events and then searched for such genes in other fishes.

BLAST searching was employed for the initial discovery analysis of putative HGT events^27^. Firstly, each protein sequence in *D. rerio* was compared with other animal sequences in the local database using BLASTP. The results were filtered based on an E-value threshold of 1e^−10^ and a continuous overlap threshold of 33% with the query protein. Because we aimed to detect HGT events in fishes, proteins with homologs in fishes only were selected as possible candidate HGTs^45^. Next, these candidates were compared to the non-animal database via BLASTP with the same threshold as above. Homologs present in non-animal species were subjected to further phylogenetic analyses to validate the reliability of HGT origins (see below). Putative horizontally transferred genes in *D. rerio* were compared with other fish proteins in the local database using BLASTP and with their genomes using TBLASTN^27^.

To eliminate potential DNA contamination, we retained candidate horizontally transferred genes present in many fishes because different kinds of fish constituted independent samples and, therefore, contamination was unlikely to involve identical genes. Further, phylogenies were generated to detect the origins and transmissions of genes adjacent to each candidate gene in the genome^48^. Finally, considering that bacteria lack introns, bacterial contamination was ruled when candidate horizontally transferred genes had introns.

### Validation of horizontal transfer

Phylogenetic analyses were employed to validate the histories of putative horizontally transferred genes. The clustering of a fish gene with a candidate donor group was assumed to support HGT from a particular donor to fish. Homologs of fish horizontally transferred genes were searched in non-redundant databases by BLASTP (E-value = 1e^−10^). Next, the homologous protein sets were submitted to multiple alignment using ClustalW2^49^. Unrooted tree-building used three approaches: Bayesian inference (BI), maximum likelihood (ML) and distance-based neighbour-joining (NJ). Prottest v3.0^50^ was used to estimate the most optimal models of amino acid substitution. WAG + I + G was the most optimal model for XP_001335286.1. BI analyses were conducted using MrBayes v3.1.2^51^. For each HGT, searches were run using four MCMC chains for one million generations with sampling every 100th tree. Searching was stopped when the average standard deviation of split sequences approached zero. The initial 2500 trees were discarded as burn-in, and a 50% majority-rule consensus tree was then constructed to obtain Bayesian posterior probabilities (BPP) for the nodes and those with BPP values greater than 95% were considered to be strongly supported. ML phylogenies were constructed by Phyml v3.1^52^. The branch support values were generated by performing 1,000 bootstrap (BS) pseudoreplicates. NJ trees were obtained using MEGA6.0^53^. The evolutionary distances for NJ construction were computed using the Poisson correction method. Nodal support was obtained by generating 1,000 BS pseudoreplicates. BS values greater than 90% were assumed to indicate strong nodal support. Resulting trees were visualized and edited using FigTree v1.3.1. To the phylogenetic analyses of eukaryotes, 150 genes that were most evenly sampled across major eukaryote clades used in previous study were download^54^ and ML phylogenies were constructed by Phyml v3.1^52^.

### Putative functional assignments

We performed COG, GO, and KEGG analyses for each HGT gene to infer its function. A putative HGT protein-coding gene was subjected to SignalP^30^, TargetP^31^, and TMHMM^32^ analyses to identify and predict its possible secretion signals and/or transmembrane domains. Secretory proteins were required to have a signal peptide (SignalP) predicted as secreted (TargetP) and no transmembrane helices (TMHMM). We used WoLFPSORT^33^ to infer cellular locations. Protein domain predictions were made using Pfam^55^ and SMART (Simple Modular Architecture Research Tool; http://smart.embl-heidelberg.de/) analyses^56^. Conserved motif analysis was performed by MEME and Weblogo using default settings^57^,^58^. Motifs with p-values less than 1e^−10^ were considered to be significant. The tertiary structures of HGT-coding proteins were determined using the SWISS-MODEL web server (http://swissmodel.expasy.org/)^59^.

### Gene expression

RNA-Seq data for sperm, oocyte, 1-cell, 4-cell, 16-cell, 64-cell, 128-cell, 256-cell, 1,000-cell and sphere of *D. rerio* were downloaded from the Gene Expression Omnibus (GEO) repository of NCBI under accession GSE44075. Raw RNA-Seq reads for each sample were stripped of their adaptor sequences with Cutadapt (http://code.google.com/p/cutadapt/) and we removed low quality bases by using Trimmomatic^60^. Reads that were less than 20 nt in length or that contained an ambiguous nucleotide were discarded. The remaining reads were aligned to *D. rerio* RefSeq genome Zv9 using TopHat2 while allowing for up to two mismatches^61^,^62^. Uniquely mapped reads with mapping qualities larger than or equal to 20 were retained for subsequent analyses. The number of reads mapped to each gene in each RNA-Seq sample was counted using the HTSeq python package (http://www-huber.embl.de/users/anders/HTSeq/doc/overview.html) with the ‘union’ overlap resolution mode and -stranded = no. For each sample, RPKM was computed as the number of reads that mapped per kilobase of exon model per million mapped reads for each gene^63^.

### GC content and codon usage

In general, GC content and patterns of codon usage in horizontally transferred genes have been used to assess the extent of amelioration. Transferred genes were deemed to have already gone through amelioration if their signature bore greater similarity to the genome of the host than the putative donor.

GC content and codon usage bias indices, including CAI (codon adaptation index), CBI (codon bias index), FOP (frequency of optimal codon) and ENC (effective number of codons), were determined for the protein-coding sequences of putative horizontally transferred genes as well as all genes of fishes and the putative donor using CodonW (http://codonw.sourceforge.net/). Non-animal species with the highest sequence identity and occurrence on the same branch in horizontally transferred gene tree was deemed as the putative donor. All coding sequences of donor were downloaded from NCBI. We compared the indices of horizontally transferred gene to the average indices of protein-coding genes of fishes and donor.

### Molecular evolution

Selection analyses identified pressures operating on horizontally transferred genes. Selection was assessed using the ML method of Codeml in PAML v4.7^35^. PAL2NAL^64^ was used to convert protein sequence alignments into the corresponding codon-based DNA alignments. First, an analysis under the one-ratio model (M0) was performed to estimate a global omega (ω) value (dN/dS ratio) across the phylogeny. Global selective pressures were assessed using site models M1a, M2a, M8, and M8a. Evolutionary rates of particular branches of interest (ω1) versus the background ratio (ω0) were computed using the branch model (model = 2). Selective pressures operating on subsets of sites of these branches were calculated using the branch-site models A and A1. The significance of change of ω and evidence of positive selection was assessed using the likelihood ratio test. Positive sites were identified using Bayes Empirical Bayes (BEB)^65^.

## Additional Information

**How to cite this article**: Sun, B.-F. *et al.* Horizontal functional gene transfer from bacteria to fishes. *Sci. Rep.* **5**, 18676; doi: 10.1038/srep18676 (2015).

## Supplementary Material

Supplementary Information
