# Supplementary material for: Horizontal functional gene transfer from bacteria to fishes
Source: Sci Rep. 2015 Dec 22;5:18676. doi: 10.1038/srep18676 (PMC4687049; doi:10.1038/srep18676)
Supplement: Supplementary Information [file srep18676-s1.pdf]

Horizontal functional gene transfer from bacteria to fishes

Bao-Fa Sun[1,2*], Tong Li[3*], Jin-Hua Xiao[1], Ling-Yi Jia[1], Li Liu[4], Peng Zhang[1], Robert W. Murphy[5], Shun-Min He[1], Da-Wei Huang[1,6 §]

List of additional files:

Additional Figure 1. Flow chart for detecting horizontally transferred gene along with the results of each step.

Additional Figure 2. Phylogenetic trees of NP_001018555.1 homologs. The ML (maximum likelihood) unrooted tree is shown. Numbers above branches indicate bootstrap support values of maximum likelihood when they are more than 50%. Scale bar indicates substitutions per site.

Additional Figure 3. Values of four indices of codon bias, CAI, codon adaptation index; CBI, codon bias index; Fop, frequency of optimal codon; ENC, effective number of codons of transferred gene in *Danio rerio* and putative donor, respectively. The red curves show values distribution of whole genes in *Danio rerio.* The blue lines indicated the values of transferred gene XP_001335286.1 in *Danio rerio*, while the green lines indicated the values of its homology WP_045030457.1 in putative donor *Draconibacterium* sp.

Additional Table 1. Homologs of XP_001335286.1 in the non-redundant (NR) database of NCBI.

Figure S1

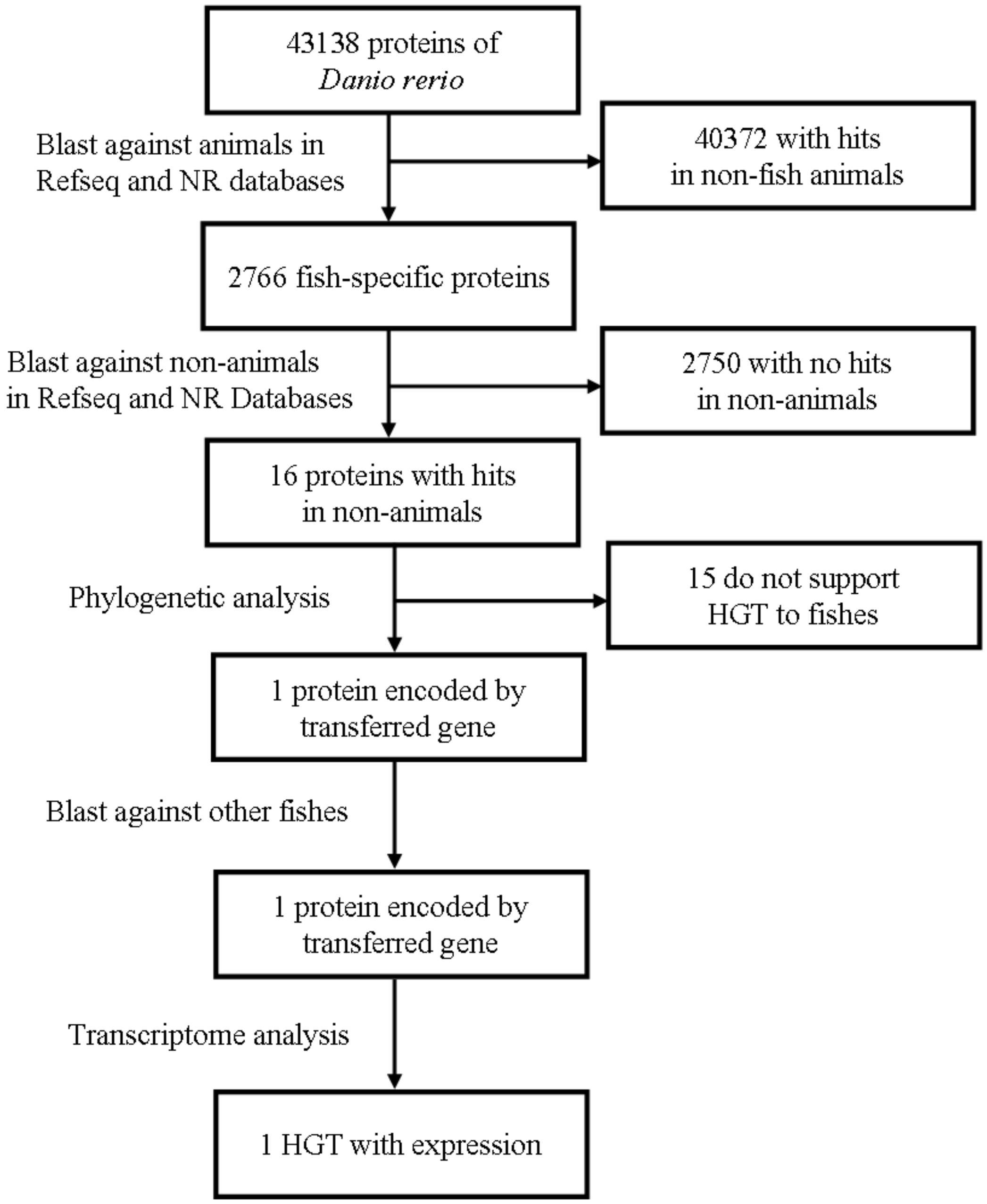
43138 proteins of
Danio rerio
Blast against animals in
Refseq and NR databases
40372 with hits
in non-fish animals
2766 fish-specific proteins
Blast against non-animals
in Refseq and NR Databases
2750 with no hits
in non-animals
16 proteins with hits
in non-animals
Phylogenetic analysis
15 do not support
HGT to fishes
1 protein encoded by
transferred gene
Blast against other fishes
1 protein encoded by
transferred gene
Transcriptome analysis
1 HGT with expression

Figure S2

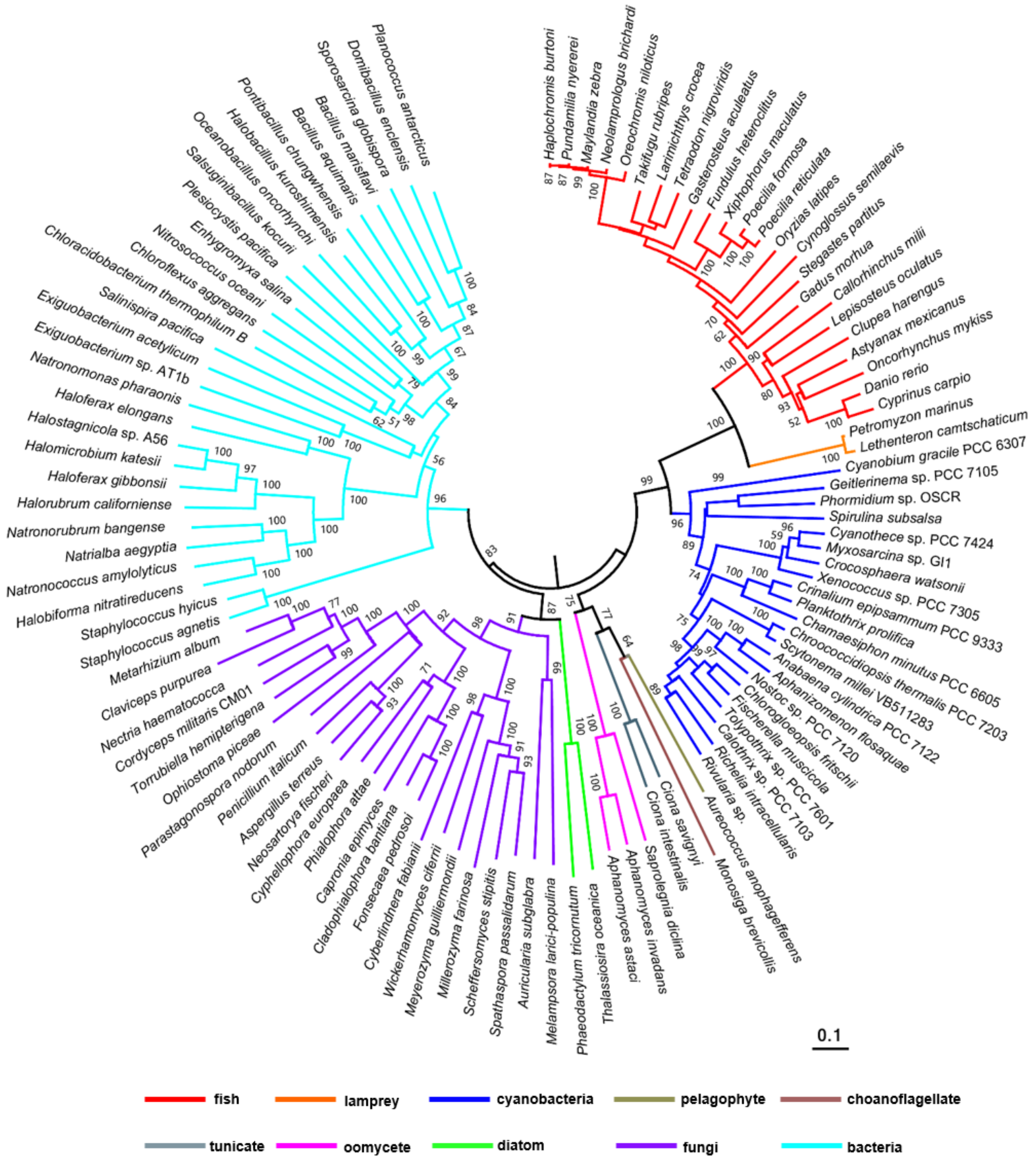
Haplochromis burtoni
Pundamilia nyererei
Maylandia zebra
Neolamprologus brichardi
Oreochromis niloticus
Takifugu rubripes
Larimichthys crocea
Tetraodon nigroviridis
Gasterosteus aculeatus
Fundulus heteroclitus
Xiphophorus maculatus
Poecilia formosa
Poecilia reticulata
Oryzias latipes
Cynoglossus semilaevis
Stegastes partitus
Gadus morhua
Callorhinchus milii
Lepisosteus oculatus
Clupea harengus
Astyanax mexicanus
Oncorhynchus mykiss
Danio rerio
Cyprinus carpio
Petromyzon marinus
Lethenteron camtschaticum
Cyanobium gracile PCC 6307
Geitlerinema sp. PCC 7105
Phormidium sp. OSCR
Spirulina subsalsa
Cyanothece sp. PCC 7424
Myxosarcina sp. GI1
Crocosphaera watsonii
Xenococcus sp. PCC 7305
Crinalium epipsammum PCC 9333
Planktothrix prolifica
Chamaesiphon minutus PCC 6605
Chroococcidiopsis thermalis PCC 7203
Scytonema millei VB511283
Anabaena cylindrica PCC 7122
Aphanizomenon flosaquae
Nostoc sp. PCC 7120
Chlorogloeopsis fritschii
Fischerella muscicola
Tolypothrix sp. PCC 7601
Calothrix sp. PCC 7103
Richelia intracellularis
Rivularia sp.
Aureococcus anophagefferens
Monosiga brevicollis
Ciona savignyi
Ciona intestinalis
Saprolegnia diclina
Aphanomyces invadans
Aphanomyces astaci
Thalassiosira oceanica
Phaeodactylum tricornutum
Melampsora larici-populina
Auricularia subglabra
Spathaspora passalidarum
Scheffersomyces stipitis
Millerozyma farinosa
Meyerozyma guilliermondii
Wickerhamomyces ciferrii
Cyberlindnera fabianii
Fonsecaea pedrosoi
Cladophialophora bantiana
Capronia epimyces
Phialophora attae
Cyphellophora europaea
Neosartorya fischeri
Aspergillus terreus
Penicillium italicum
Parastagonospora nodorum
Ophiostoma piceae
Torrubiella hemipterigena
Cordyceps militaris CM01
Nectria haematococca
Claviceps purpurea
Metarhizium album
Staphylococcus agnetis
Staphylococcus hyicus
Halobiforma nitratireducens
Natronococcus amylolyticus
Natrialba aegyptia
Natronorubrum bangense
Halorubrum californiense
Haloferax gibbonsii
Halomicrobium katesii
Halostagnicola sp. A56
Haloferax elongans
Natronomonas pharaonis
Exiguobacterium sp. AT1b
Exiguobacterium acetylicum
Salinispira pacifica
Chloracidobacterium thermophilum B
Chloroflexus aggregans
Nitrosococcus oceani
Enhygromyxa salina
Plesiocystis pacifica
Salsuginibacillus kocurii
Oceanobacillus oncorhynchi
Halobacillus kuroshimensis
Pontibacillus chungwhensis
Bacillus aquimaris
Bacillus marisflavi
Sporosarcina globispora
Domibacillus enclensis
Planococcus antarcticus
0.1
fish
lamprey
cyanobacteria
pelagophyte
choanoflagellate
tunicate
oomycete
diatom
fungi
bacteria

Figure S3

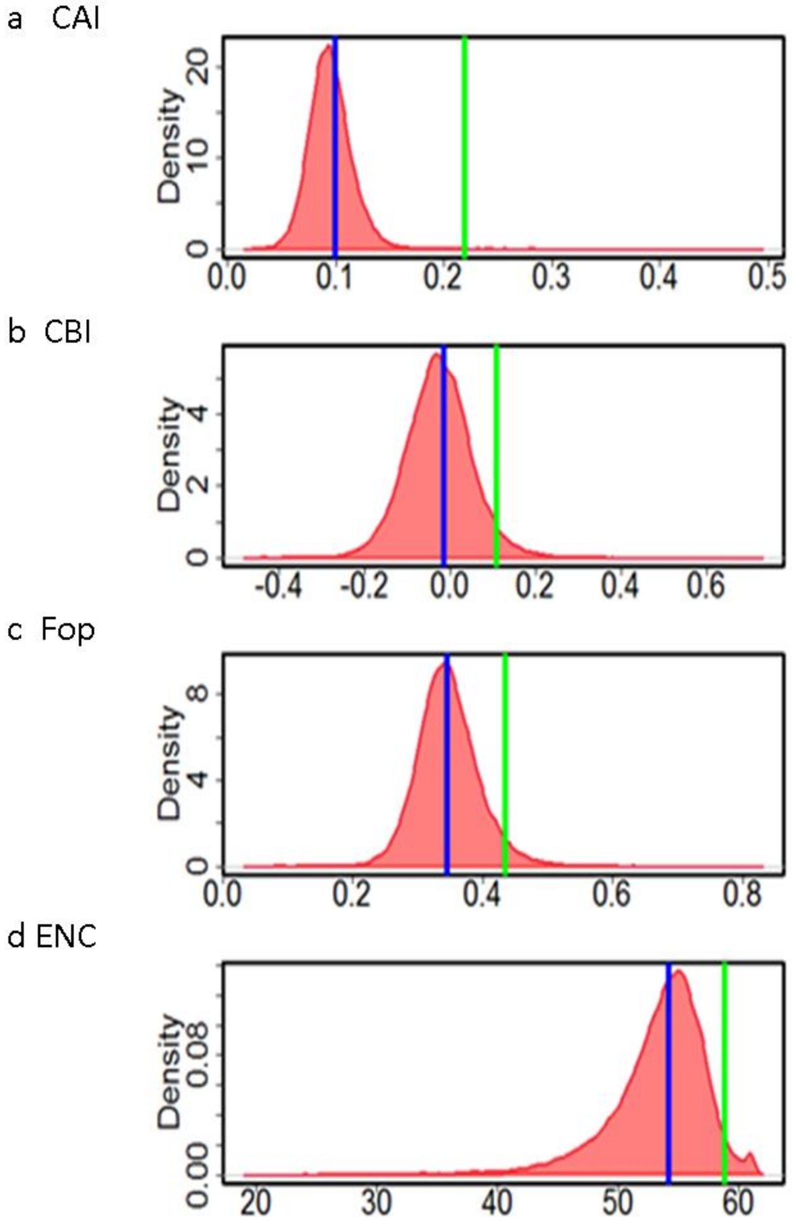
a CAI
Density
0
10
20
0.0
0.1
0.2
0.3
0.4
0.5
b CBI
Density
0
2
4
-0.4
-0.2
0.0
0.2
0.4
0.6
c Fop
Density
0
4
8
0.0
0.2
0.4
0.6
0.8
d ENC
Density
0.00
0.08
20
30
40
50
60

Table S1. Homologs of XP_001335286.1 in the non-redundant (NR) database of NCBI. Names with red font are the top hit species apart from fishes with homologs of transferred gene in *Danio rerio*. *Draconibacterium* sp. is a marine bacterium.

Species	ID	Similarity	Length	No. Exons
Danio rerio	XP_001335286.1	100%	322	2
Oreochromis niloticus	XP_005455187.1	78%	320	3
Poecilia reticulata	XP_008429143.1	77%	320	3
Larimichthys crocea	XP_010750882.1	77%	320	2
Neolamprologus brichardi	XP_006791882.1	77%	320	3
Pundamilia nyererei	XP_005739302.1	77%	320	3
Maylandia zebra	XP_004560234.1	77%	320	3
Haplochromis burtoni	XP_005929494.1	77%	320	3
Astyanax mexicanus	XP_007239793.1	77%	263	3
Poecilia Formosa	XP_007569943.1	76%	320	3
Stegastes partitus	XP_008285582.1	76%	320	2
Xiphophorus maculatus	XP_005814593.1	75%	320	2
Oncorhynchus mykiss	CDQ78821.1	74%	319	2
Cynoglossus semilaevis	XP_008322373.1	74%	320	3
Takifugu rubripes	XP_003977721.1	73%	322	2
Tetraodon nigroviridis	CAG04555.1	71%	322	2
Draconibacterium sp.	WP_045030457.1	58%	326	1
